# Developing family medicine in Zambia

**DOI:** 10.4102/phcfm.v7i1.909

**Published:** 2015-10-30

**Authors:** Mpundu Makasa, Selestine Nzala, Jim Sanders

**Affiliations:** 1 Department of Public Health, Community and Family Medicine Unit, School of Medicine, University of Zambia, Lusaka, Zambia; 2 Department of Family and Community Medicine, Medical College of Wisconsin, Madison, United States

Zambia is classified as a low-income country and has a population of 13.2 million people, of whom 51% reside in rural areas.^1^ There is a double burden of disease and, whilst the country still grapples with communicable diseases such as HIV and/or AIDS, tuberculosis and malaria, there is a growing problem of non-communicable diseases.^2^

There are approximately 1400 facilities in the country’s health infrastructure, of which 85% are in the public sector and the remainder are either in the private or faith-based sectors.^3^ The country faces a human resource crisis and has only about 1200 doctors,^4^ which gives a ratio of 0.09 doctors per 1000 population, falling far short of the World Health Organization’s recommendation of at least 2.3 physicians per 1000, which would be sufficient to achieve coverage for primary healthcare.^5^ Only about 650 doctors are in the public sector,^6^ of whom the majority reside and work in urban areas,^4^^,6^ which means that the disadvantaged rural areas depend on other healthcare providers, such as nurses and clinical officers, to provide services. These providers are able to provide basic care, but more advanced or complicated care is outside their scope of practice.

Zambia’s healthcare system has a three-tier structure: primary, secondary and tertiary levels. The primary level consists of health posts, health centres and district hospitals. It is envisioned that family physicians, being expert generalists, will work in district hospitals and be lead clinicians overseeing the primary level of care.

The University of Zambia’s School of Medicine recognises its strategic importance in responding to and addressing the country’s human resource needs. Part of this response includes the introduction of a postgraduate training programme in Family Medicine. It is hoped that family physicians, who are expert generalists in primary care, can strengthen primary healthcare and redress some of the inequity in human resources by working in rural, remote and underserved community-based settings. In Zambia, family physicians will be specifically trained to provide clinical leadership within district health services, a model seen in other countries in the region. A critical mass of family physicians working within the district health services should lead to a reduction in cases referred to higher levels of care, and ultimately to a reduction in morbidity and mortality.

To achieve these reductions, a working group was constituted to spearhead the development of postgraduate training in Family Medicine at the University of Zambia. Some important steps taken include engaging stakeholders and obtaining their support and involvement. Key stakeholders include the Ministry of Health and Ministry of Community Development, Mother and Child Health, the Health Professions Council of Zambia, School of Medicine senior faculty, senior members of the medical fraternity and selected Zambian family physicians, who were all foreign-trained and in private practice in Lusaka. The key stakeholders have so far all pledged to support the new programme, and a common understanding of the roles and the career progression of the family physician were arrived at.

A Zambian family physician was defined as a primary healthcare specialist who provides holistic clinical and preventive care, healthcare management and research. Similar to other specialist programmes, the residents would progress from registrar to senior registrar upon completion, and to consultant level. It is envisaged that the Ministry of Health through its commitment will advocate for positions for these specialists on its establishment. Currently, the Health Professions Council of Zambia recognises doctors trained in this area and has a specialist register for family physicians.

A major milestone has been the development of a curriculum based on principles of health equity, which reflects the health needs of Zambia. The programme will be established at the Department of Public Health within the School of Medicine and was expected to start in September 2015. Immediate plans include expediting the adoption of the curriculum, recruitment and acceptance of trainees, securing training materials, and increasing and developing the faculty.

Finally, the department places much emphasis on fostering partnerships and the need to network with countries within the region and elsewhere who are already training family physicians.
